# Performance of large language models in reporting oral health concerns and side effects in head and neck cancer: a comparative study

**DOI:** 10.1007/s00432-025-06400-w

**Published:** 2025-12-20

**Authors:** Jonas Rast, Susanne Wiegand, Jana Biermann, Annette Wiegand, Felix Marschner

**Affiliations:** 1 Department of Otorhinolaryngology, Head and Neck Surgery, University Hospital Schleswig-Holstein, Arnold-Heller-Straße 3, 24105 Kiel, Germany; 2 Department of Preventive Dentistry, Periodontology and Cariology, University Medical Center Göttingen, Robert-Koch-Str. 40, 37075 Göttingen, Germany

**Keywords:** Head and neck cancer, Oral health, Artificial intelligence, Large language model, Readability, Information quality

## Abstract

**Purpose:**

With increasing reliance on large language models (LLMs) for health information, this study evaluated reliability and quality, understandability, actionability, readability and misinformation risk of responses from LLMs to oral health concerns and oral side effects in head and neck cancer (HNC) patients.

**Methods:**

Frequently asked questions on oral health and HNC therapy side effects were identified via ChatGPT–GPT-4-turbo and Gemini–2.5 Flash, then submitted to eight LLMs (ChatGPT–GPT-4-turbo, Gemini–2.5 Flash, Microsoft Copilot, Perplexity, Chatsonic, Mistral, Meta AI–Llama 4, DeepSeek–R1). Responses were assessed using DISCERN and modified DISCERN instruments (reliability and quality), Patient Education Materials Assessment Tool (PEMAT [understandability and actionability]), Flesch-Reading-Ease-Score (FRES [readability]), misinformation score, citations, and wordcounts. Statistical analysis was done by Scheirer-Ray-Hare-test followed by Dunn’s post-hoc-tests and Bonferroni-Holm correction (*p* < 0.05).

**Results:**

A total of 40 questions belonging to 12 oral health-related categories were identified. Statistically significant differences between LLMs were found for DISCERN, modified DISCERN, PEMAT-understandability, PEMAT-actionability, FRES, and word counts (*p* < 0.001). Median DISCERN and modified DISCERN scores amounted from 47.0 (ChatGPT–GPT-4-turbo) to 59.0 (Perplexity, Chatsonic) and from 2.0 (Gemini–2.5 Flash, Mistral) to 5.0 (Perplexity) indicating good to fair reliability. LLMs were understandable (median PEMAT-understandability scores ≥ 75.0), but provided limited specific guidance (median PEMAT-actionability scores ≤ 40) and used complex language (median FRES ≤ 40.2). Misinformation risk was generally low and not statistically significant among LLMs (*p* = 0.768).

**Conclusion:**

Despite a low overall misinformation risk, deficits in actionability highlight the need for cautious integration of LLMs into HNC patient education.

**Supplementary Information:**

The online version contains supplementary material available at 10.1007/s00432-025-06400-w.

## Introduction

Patients with head and neck cancer (HNC) often face considerable treatment-related oral health challenges, including xerostomia, mucositis, radiation-induced dental caries, and osteoradionecrosis (Bhandari et al. 2020; Gouvêa Vasconcellos et al. 2020; Wadhawan et al. 2025). These conditions can significantly influence oral health-related quality of life and reduce adherence to treatment (Epstein et al. 2012; Rose et al. 2025).

Informing patients plays a crucial role in enhancing their understanding of health conditions and preventive strategies, thereby contributing to improved adherence to therapy (Jabbour et al. 2022). Among patients with HNC, the internet ranks as the second most used source of information, following direct consultations with healthcare professionals (Jabbour et al. 2017). With people increasingly relying on the internet for health-related information, web-based patient education materials have become increasingly popular (Sun et al. 2023). As a result, access to accurate, understandable, and actionable health information is essential for these patients (Sun et al. 2023).

In this context, large language models (LLMs) have emerged as accessible tools for health-related information (Meyrowitsch et al. 2023; Qiu et al. 2023). LLMs are artificial intelligence systems trained on textual data from articles, websites, and other digital sources to understand, interpret, and generate human language, enabling them to communicate much like humans do (Vedula et al. 2022; Eggmann et al. 2023). Based on this data, they can produce context-aware replies, which are applied in various fields such as healthcare communication, education, and customer service (Biswas 2023). In recent years, LLMs such as ChatGPT (OpenAI) and Gemini (Google) have emerged as accessible tools for health-related information retrieval. LLMs have the potential to support patient education by providing timely and personalized responses to complex medical questions. While LLMs may have various useful applications, they come with risks of malicious use and serious limitations, including the potential for misinformation (Eggmann et al. 2023). Moreover, recent findings suggest that even minimal data corruption during LLM training can cause harmful misinformation, such as recommending non–evidence-based alternative cancer treatments or misrepresenting side effects and symptoms (Hryciw et al. 2023). Patients unaware of the limitations of LLMs, including the risk of misinformation or failure to account for individual differences, like gender and ethnicity may trust LLMs over physicians, potentially undermining confidence, assumptions, and treatment adherence (Derevianko et al. 2023; Hryciw et al. 2023; Omiye et al. 2023). However, the reliability and quality, understandability, actionability, readability, and risk of misinformation in LLM-generated content are still being closely examined, especially in sensitive areas like cancer supportive care. A recent study indicated that ChatGPT provides moderately accurate information on oropharyngeal cancer, but it may misinform patients and often uses linguistically complex language (Davis et al. 2024). While studies have evaluated the quality of artificial intelligence generated responses for common types of cancer such as skin, lung, breast, colorectal, and prostate cancer (Pan et al. 2023; Yeo et al. 2023; Hershenhouse et al. 2025; Park et al. 2025), there is currently no comprehensive analysis assessing the quality of responses to frequently asked questions about oral health and treatment-related oral side effects in HNC in different common LLMs.

The aim of this comparative study is to evaluate the quality, understandability, actionability, risk of misinformation, and accuracy of responses provided by eight publicly available LLMs: ChatGPT–GPT-4-turbo (OpenAI), Gemini–2.5 Flash (Google) Microsoft Copilot (Microsoft), Perplexity (Perplexity.AI), Chatsonic (Writesonic), Mistral (Mistral AI), and Meta AI–Llama 4 (Meta Platforms), and DeepSeek–R1 (Hangzhou DeepSeek Artificial Intelligence Basic Technology Research) to frequently asked questions about oral health and therapy-related oral side effects in patients with HNC.

## Methods

The following study adhered to the Enhancing Transparency in Reporting the Synthesis of Qualitative Research guidelines (Tong et al. 2012), and to the Reporting Guideline for Chatbot Health Advice Studies (Huo et al. 2025). This study used only publicly available data.

### Data sources

As no information on the top 10 questions related to oral health and side effects in HNC were available, data were obtained directly from two common LLMs: ChatGPT–GPT-4-turbo (release date: November 2023) and Gemini–2.5 Flash (release date: April 2025). Two examiners (J.R. and F.M.) independently submitted the following prompt on June 2, 2025, via web browser from Kiel and Göttingen, Germany: “What are the 10 most frequently asked questions on oral health and oral side effects of head and neck cancer treatment?” [original prompt in German: Welche 10 Fragen werden am häufigsten zur Mundgesundheit und zu oralen Nebenwirkungen bei der Therapie bei Kopf-Hals-Tumoren gestellt? ], to identify the most frequently asked questions on this topic. After receiving the responses, a list of 40 questions (10 questions provided by each examiner for each of the two LLMs) was compiled and served as the basis for further analysis (Supplemental Table S1). The examiners were not blinded to the LLMs used.

In the second phase of the study, the 40 patient questions were entered into eight commonly used LLMs between June 05 and 12, 2025, also via web browser from Kiel and Göttingen, Germany: ChatGPT–GPT-4-turbo (release date: November 2023), Gemini–2.5 Flash Microsoft (release date: April 2025), Copilot (release date: March 2023), Perplexity (release date: December 2022), Chatsonic (release date: January 2021), Mistral (release date: December 2023), Meta AI–Llama 4 (release date: April 2025), and DeepSeek–R1 (release date: January 2025). For all questions, model-specific default settings were applied, and new conversations were initiated. All LLMs evaluated in this study were base models without additional fine-tuning. Meta AI–Llama 4 and Mistral are open-source LLMs, while all other LLMs used in this study are closed-source LLMs. All prompts were formulated in German language. Prompts were standardized and not iteratively modified. No patients or laypersons were involved in prompt development. The LLM responses were transferred to Microsoft Word 365 (Microsoft Corporation) for readability analysis, following previous studies (Gül et al. 2024; Hancı et al. 2024).

## Outcomes

The following outcomes were assessed:

1. reliability and quality were assessed using the DISCERN instrument, which consists of 16 questions evaluating the reliability of the source, the quality of treatment-related content, as well as the transparency and balance of the presented information, and provides an overall rating. Each question is scored on a 5-point scale from 1 (no) to 5 (yes) (Charnock et al. 1999) (total score range: 16 to 80, with higher scores indicating better reliability). Additionally, a modified version of the DISCERN instrument was applied (Uzun 2023). The modified DISCERN instrument includes 5 questions from the DISCERN instrument. It covers aims and clarity, use of reliable sources, balance and bias, additional sources, and areas of uncertainty, with each item scored 0 or 1 point (score range: 0 to 5, with higher scores indicating better reliability);
2. understandability and actionability using the Patient Education Materials Assessment Tool (PEMAT) (Shoemaker et al. 2020). PEMAT assesses how easily patient education materials can be understood and applied. It contains 24 questions, each is scored as agree (1), disagree (0), or not applicable (N/A). Separate percentage scores are calculated for understandability and actionability by dividing the total points earned by the maximum possible points, excluding any items marked as N/A (score range: 0% to 100%, with higher scores indicating greater understandability and actionability);
3. readability using the Flesch Reading Ease Score (FRES) (Flesch 1948), adapted by Amstad for the German language (Amstad 1978). The FRES evaluates the linguistic difficulty of a text by considering the average sentence length (in words), and the average number of syllables per word (score range: 0 to 100, with higher scores indicating easier-to-read text);
4. the risk of misinformation using a 5-point Likert scale (Loeb et al. 2019) (score range: 1 to 5, with higher scores indicating lower risk of misinformation), matched against the guidelines of the European Head and Neck Society, the European Society for Medical Oncology, the European Society for Radiotherapy and Oncology, the International Society of Oral Oncology, the Multinational Association of Supportive Care in Cancer, the American Society of Clinical Oncology and the National Comprehensive Cancer Network (Nekhlyudov et al. 2017; Elad et al. 2020; Machiels et al. 2020; Pfister et al. 2020; Watson et al. 2021; Doctor et al. 2024; Peterson et al. 2024; Colevas et al. 2025). A response was scored as misinformation if it contradicted the recommendations in these guidelines. Information from LLMs, mentioned only in the general context of the guidelines, was evaluated using the standardized clinical procedures at both clinics;
5. number of citations from LLMs responses, and word counts.

Outcomes were independently evaluated by two examiners (J.R. and F.M.). Interrater reliability was calculated based on the independent evaluations. In cases of disagreement, consensus was reached through discussion for the dataset used for statistical analysis. FRES and word counts were calculated using a freely available online tool (Schöll 2025), and evaluated by a single examiner (F.M.).

### Statistical analysis

Categorical data are presented as counts (n) and percentages (%), and continuous data as medians with interquartile ranges, and range. Kolmogorov-Smirnov and Shapiro-Wilk tests were applied to check normal distribution of the data. As data were not normally distributed, Scheirer-Ray-Hare test (a non-parametric two-way ANOVA on ranks) (Scheirer et al. 1976) followed by Dunn’s post-hoc tests with Bonferroni-Holm correction were applied.

For all outcome parameters except FRES and word counts interrater reliability among examiners was assessed using intraclass correlation coefficient (ICC 2,1) analysis (Shrout and Fleiss 1979). Statistical analyses were performed using R version 4.5.1 (www.r-project.org). The level of significance was set at *p* < 0.05.

## Results

The most frequently asked questions presented by both LLMs (ChatGPT–GPT-4-turbo and Gemini–2.5 Flash) had slightly different text but allowed sorting the data into 12 categories: dental and oral hygiene (*n* = 8), side effects (*n* = 6), dental care (*n* = 4), xerostomia (*n* = 4), dental complications (*n* = 3), gustatory dysfunction (*n* = 3), mucositis (*n* = 3), nutrition (*n* = 3), oral pain (*n* = 3), dysphagia (*n* = 1), oral candidiasis (*n* = 1), and osteoradionecrosis (*n* = 1). Further information is provided in Supplemental Table S1.

**Table 1:** Main and interaction effects of large Language models and categories across outcomes in oral health-related questions

Outcome	Effect	η²	*p*-value
DISCERN score	LLM	0.359	< 0.001*
	Category	0.081	0.007*
	LLM, Category	0.225	0.652
Modified DISCERN score	LLM	0.597	< 0.001*
	Category	0.018	0.885
	LLM, Category	0.143	0.998
PEMAT understandability score	LLM	0.268	< 0.001*
	Category	0.101	< 0.001*
	LLM, Category	0.209	0.797
PEMAT actionability score	LLM	0.136	< 0.001*
	Category	0.217	< 0.001*
	LLM, Category	0.255	0.350
FRES for German language	LLM	0.315	< 0.001*
	Category	0.209	< 0.001*
	LLM, Category	0.151	0.996
Misinformation score	LLM	0.013	0.768
	Category	0.038	0.352
	LLM, Category	0.404	< 0.001*
Citations	LLM	0.804	< 0.001*
	Category	0.009	0.994
	LLM, Category	0.054	1.000
Word counts	LLM	0.651	< 0.001*
	Category	0.055	0.098
	LLM, Category	0.112	1.000

LLM: Large language model; PEMAT: Patient Education Materials Assessment Tool; FRES: Flesch Reading Ease Score; η²: effect size; *, statistical significance (*p* < 0.05)

**Fig. 1:**
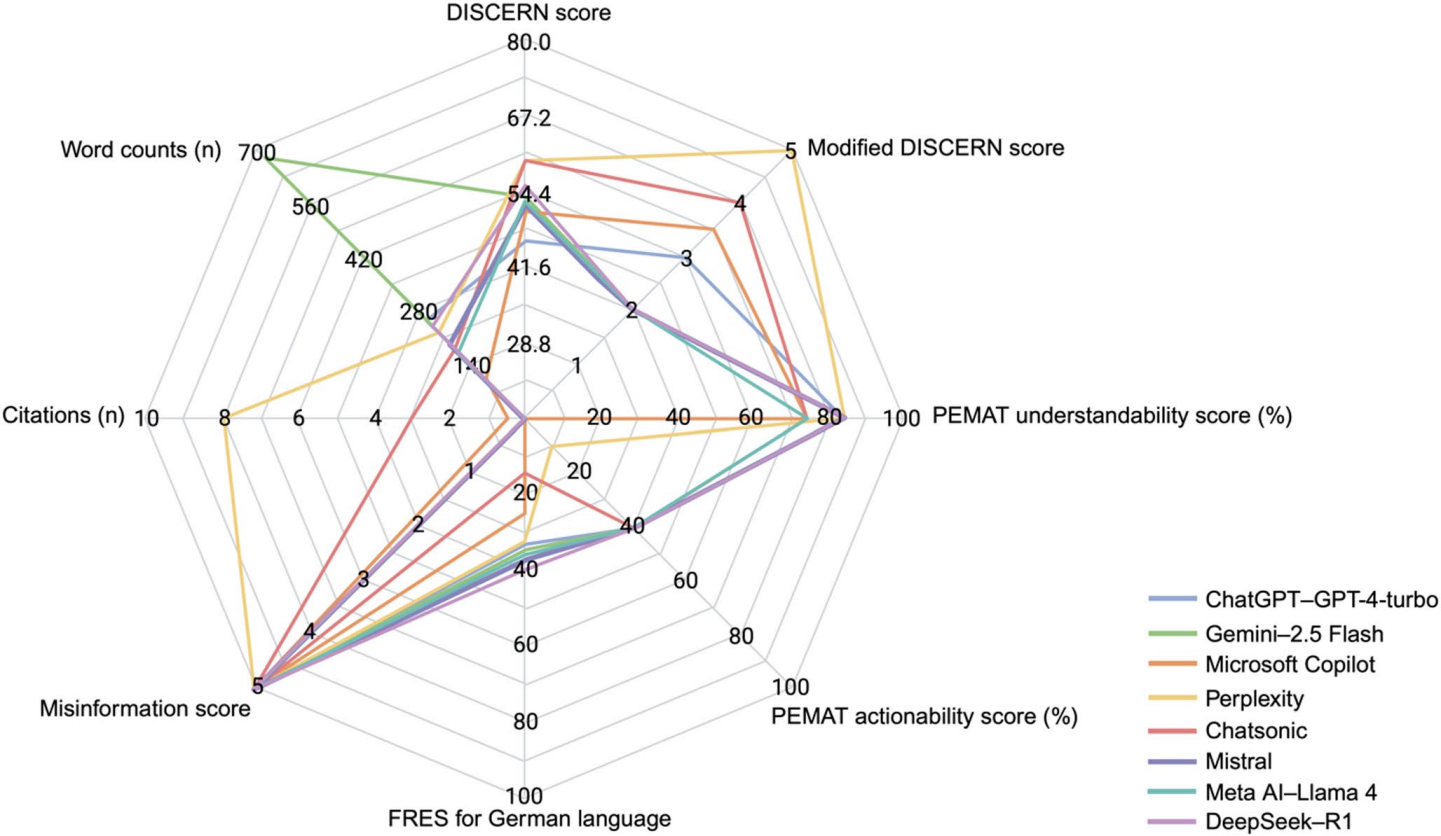
Radar chart of median outcomes of large language models. PEMAT: Patient Education Materials Assessment Tool; FRES: Flesch Reading Ease Score; created in https://BioRender.com

Except for misinformation score, all outcomes were significantly affected by the kind of LLM used (*p* < 0.001, Table 1). DISCERN and modified DISCERN scores were significantly different among groups, with Chatsonic and Perplexity outperforming most of the other LLMs. PEMAT understandability scores were generally high and better for ChatGPT–GPT-4-turbo, Perplexity, Gemini–2.5 Flash, DeepSeek–R1 and Mistral than for Microsoft Copilot. The PEMAT actionability score was comparatively low, with Microsoft Copilot performing significantly worse compared to all other LLMs. Regarding overall FRES, LLM generated responses were difficult to read (Weiss 2003). Among the evaluated LLMs, Chatsonic and Microsoft Copilot performed worst compared to almost all others (Fig. 1). All LLMs demonstrated a low risk of misinformation, with an overall misinformation score of 5.0 (range: 4–5). Perplexity referred to significantly most citations, followed by Chatsonic, Microsoft Copilot, and Gemini–2.5 Flash, while the remaining LLMs displayed no citations. If citations were included, they referred to agencies, hospital-affiliated institutions, independent volunteer health organizations, online encyclopedias, manufacturers of oral care products, and medical websites. Responses of LLMs ranged from 55 to 1541 words, with Gemini–2.5 Flash generating the significantly longest response, followed by ChatGPT–GPT-4-turbo and DeepSeek–R1. Further information is provided in Table 2. Interrater reliability was good to excellent for all outcomes (Table 3).

**Table 2:** Performance of large Language models on oral health and treatment-related side effect queries in head and neck cancer

Outcome median (IQR) range	All LLMs	Large language models							
		ChatGPT–GPT-4-turbo	Gemini–2.5 Flash	Microsoft Copilot	Perplexity	Chatsonic	Mistral	Meta AI–Llama 4	DeepSeek–R1
DISCERN score^a^	54.0 (6.0) 38–68	47.0 (8.5)^D^ 40–62	54.0 (5.0)^BC^ 38–60	51.0 (6.3)^CD^ 43–59	59.0 (5.5)^A^ 48–68	59.0 (5.0)^A^ 42–65	52.5 (4.0)^CD^ 45–58	53.0 (6.0)^BCD^ 42–62	55.0 (2.3)^AB^ 46–60
Modified DISCERN score^b^	3.0 (2.0) 0–5	3.0 (1.0)^B^ 2–4	2.0 (1.0)^B^ 1–4	3.5 (2.0)^A^ 3–5	5.0 (1.0)^A^ 4–5	4.0 (1.0)^A^ 2–5	2.0 (0.0)^B^ 0–3	2.0 (1.0)^B^ 1–5	2.0 (1.0)^B^ 2–3
PEMAT understandability score^c^ (%)	83.3 (8.3) 50.0-93.3	83.3 (8.3)^AB^ 66.7–91.7	83.3 (8.4)^A^ 50.0-91.7	75.0 (0.0)^C^ 75.0-83.3	83.3 (8.4)^AB^ 50.0-93.3	75.0 (8.3)^BC^ 58.3–91.7	83.3 (8.3)^AB^ 75.0-83.3	75.0 (8.3)^BC^ 50.0-83.3	83.3 (8.3)^AB^ 50.0-91.7
PEMAT actionability score^c^ (%)	40.0 (40.0) 0–60.0	40.0 (40.0)^A^ 0–60.0	40.0 (60.0)^A^ 0–60.0	0 (0)^B^ 0–40.0	10.0 (45.0)^A^ 0–60.0	40.0 (40.0)^A^ 0–60.0	40.0 (60.0)^A^ 0–60.0	40.0 (40.0)^A^ 0–60.0	40.0 (60.0)^A^ 0–60.0
FRES for German language^d^	34.5 (17.0) 0–59	33.8 (13.8)^A^ 2–58	35.5 (12.5)^A^ 15–52	26.0 (11.3)^BC^ 1–39	33.0 (9.3)^AB^ 18–48	16.6 (14.8)^C^ 0–52	38.6 (14.3)^A^ 5–55	37.5 (16.3)^A^ 0–59	40.2 (9.5)^A^ 18–55
Misinformation score^e^	5.0 (0) 4–5	5.0 (0) 4–5	5.0 (0) 4–5	5.0 (0) 4–5	5.0 (0) 4–5	5.0 (0) 4–5	5.0 (0) 4–5	5.0 (0) 5–5	5.0 (0) 5–5
Citations (n)	0 (2) 0–9	0 (0)^D^ 0–0	0 (0)^D^ 0–8	0.5 (2)^C^ 0–4	8 (0)^A^ 8–9	3 (1)^B^ 0–3	0 (0)^D^ 0–0	0 (0)^D^ 0–0	0 (0)^D^ 0–0
Word counts (n)	216 (114) 55-1541	269 (59)^B^ 192–376	672 (189)^A^ 73-1541	116 (28)^F^ 81–173	215 (87)^BCD^ 105–305	186 (89)^DE^ 55–310	203 (37)^CDE^ 150–273	170 (58)^EF^ 65–269	263 (82)^BC^ 77–568

IQR: interquartile range; LLMs: Large language models; PEMAT: Patient Education Materials Assessment Tool; FRES: Flesch Reading Ease Score; a: score range: 16 to 80, with higher scores indicating better reliability; b: score range: 0 to 5, with higher scores indicating better reliability; c: score range: 0% to 100%, with higher scores indicating greater understandability and actionability; d: score range: 0 to 100, with higher scores indicating easier-to-read text; e: score range: 1 to 5, with higher scores indicating lower risk of misinformation; within each row different capital letters indicate significant differences between LLMs

**Table 3:** Interrater reliability of examiners

Outcome	Intraclass correlation coefficient (2,1) (Shrout and Fleiss 1979)	95% Confidence Interval	Interpretation (Cicchetti 1994)
DISCERN score	0.996	0.995–0.997	Excellent
Modified DISCERN score	0.989	0.986–0.991	Excellent
PEMAT understandability score	0.975	0.969–0.980	Excellent
PEMAT actionability score	0.994	0.993–0.995	Excellent
Misinformation score	0.773	0.725–0.814	Good
Citations	0.980	0.975–0.984	Excellent

PEMAT: Patient Education Materials Assessment Tool

DISCERN, PEMAT understandability and actionability scores, and FRES were significantly affected by the category (Table 1). However, significant differences between categories were only found for PEMAT understandability and actionability, and FRES (Table 4). While PEMAT-understandability was comparable high across most categories, PEMAT actionability was best for dental and oral hygiene and worse for side effects. FRES indicated generally difficult readability with categories particularly challenging (e.g., side effect, dysphagia, osteoradionecrosis).

**Table 4:** Performance of large Language models on oral health and treatment-related side effect queries in head and neck cancer per category

Category	Outcome: Median (IQR)/Range							
	DISCERN score^a^	Modified DISCERN score^b^	PEMAT understandability score^c^ (%)	PEMAT actionability score^c^ (%)	FRES for German language^d^	Misinformation score^e^	Citations (*n*)	Word counts (*n*)
Dental and oral hygiene	55.0 (6.0) 43–64	2.5 (2.0) 2–5	83.3 (16.7)^A^ 66.7–91.7	40.0 (60.0)^A^ 0–60.0	37.0 (15.3)^A^ 0–51	5.0 (0) 4–5	0 (0.3) 0–8	247 (104) 90–792
Side effect	54.0 (8.3) 42–63	3.0 (2.0) 2–5	75.0 (8.3)^B^ 50.0-83.3	0 (0)^C^ 0–40.0	24.0 (13.3)^B^ 0–46	5.0 (0) 4–5	0 (2.3) 0–9	221 (99) 106–886
Dental care	56.0 (6.5) 38–62	3.0 (1.0) 0–5	83.3 (8.3)^AB^ 50.0-91.7	0 (40.0)^BC^ 0–60.0	29.5 (14.3)^AB^ 0–55	5.0 (0) 5–5	0 (1.3) 0–8	187 (99) 55–619
Xerostomia	54.0 (6.5) 40–63	3.0 (1.5) 2–5	83.3 (8.3)^AB^ 75.0-91.7	40.0 (25.0)^A^ 0–60.0	38.5 (9.5)^A^ 10–59	5.0 (0) 4–5	0 (0.5) 0–8	199 (84) 86–883
Dental complications	56.0 (7.0) 42–60	3.0 (2.0) 1–5	83.3 (8.3)^AB^ 50.0-91.7	20.0 (40.0)^ABC^ 0–60.0	37.0 (5.0)^A^ 16–50	5.0 (0) 5–5	0 (1.3) 0–8	218 (117) 65–697
Gustatory dysfunction	51.5 (6.05) 46–64	3.0 (2.3) 0–5	83.3 (8.3)^AB^ 50.0-91.7	0 (45.0)^ABC^ 0–60.0	39.5 (16.0)^A^ 17–52	5.0 (0) 4–5	0 (3) 0–8	208 (123) 124–1541
Mucositis	55.0 (5.5) 42–63	3.0 (2.3) 2–5	83.3 (8.3)^AB^ 66.7–83.3	40.0 (60.0)^AB^ 0–60.0	33.0(18.8)^A^ 0–51	5.0 (0) 5–5	0 (2) 0–8	227 (105) 112–953
Nutrition	51.0 (6.8) 45–65	3.0 (2.0) 2–5	75.0 (8.3)^AB^ 66.7–91.7	0 (40.0)^ABC^ 0–60.0	36.0 (13.5)^A^ 0–52	5.0 (0) 5–5	0 (0.3) 0–8	195 (132) 110–702
Oral pain	55.0 (7.0) 38–68	3.0 (2.0) 1–5	83.3 (8.3)^AB^ 50.0-93.3	40.0 (40.0)^AB^ 0–60.0	35.0 (16.0)^A^ 0–55	5.0 (0) 5–5	0 (1) 0–8	222 (109) 55-1021
Dysphagia	52.0 (8.0) 46–62	3.0 (1.0) 2–5	83.3 (4.2)^AB^ 75.0-91.7	40.0 (40.0)^ABC^ 0–40.0	27.0 (6.3)^AB^ 17–38	5.0 (0) 5–5	0 (0.5) 0–8	161 (176) 77–574
Oral candidiasis	56.5 (5.0) 49–60	3.0 (0.5) 2–5	83.3 (2.1)^AB^ 75.0-91.7	40.0 (0)^AB^ 20.0–60.0	39.5 (14.5)^AB^ 6–49	5.0 (0) 4–5	0 (0.8) 0–8	200 (86) 116–641
Osteoradionecrosis	58.0 (4.8) 53–68	3.0 (3.0) 2–5	83.3 (8.3)^AB^ 75.0-93.3	25.0 (40.0)^ABC^ 0–40.0	27.0 (8.8)^AB^ 4–35	5.0 (0) 4–5	0 (1) 0–8	249 (39) 128–737

IQR: interquartile range; PEMAT: Patient Education Materials Assessment Tool; FRES: Flesch Reading Ease Score; a: score range: 16 to 80, with higher scores indicating better reliability; b: score range: 0 to 5, with higher scores indicating better reliability; c: score range: 0% to 100%, with higher scores indicating greater understandability and actionability; d: score range: 0 to 100, with higher scores indicating easier-to-read text; e: score range: 1 to 5, with higher scores indicating lower risk of misinformation; within each column different capital letters indicate significant differences between categories

## Discussion

The current study aimed to assess reliability and quality, understandability and actionability, readability, and the risk of misinformation for eight common LLMs using frequently asked questions on oral health and therapy-related oral side effects in patients with HNC.

Considering the thresholds of previous studies (Weil et al. 2014; Cassidy and Baker 2016; Pradhan et al. 2025), the reliability and quality of the LLMs can be considered as good (DISCERN score: 51 to 62) to fair (DISCERN score: 39 to 50) indicating that LLMs generally can provide reliable health information, with some variations across LLMs. All tested LLMs were understandable, exceeding the benchmark of 70% (Shoemaker et al. 2020; Mani et al. 2021; Shan et al. 2023), but provided limited specific guidance (median PEMAT actionability score: 40.0%) and used complex language.

While the DISCERN score captures overall reliability and quality, the modified DISCERN specifically emphasizes key aspects of reliability and quality. Positively, most of the investigated LLMs achieved good reliability and quality. Our findings are in accordance with previous studies on LLMs (Pan et al. 2023; Hancı et al. 2024). Beyond reliability and quality, our findings, consistent with earlier research, show that PEMAT actionability scores were consistently lower than understandability scores (Pan et al. 2023; Sivaramakrishnan et al. 2025; Türe et al. 2025). LLMs tend to deliver informative content rather than concrete actionable recommendations. This suggests that LLMs are currently not reliable for actionable health guidance. Improving actionability remains the key area for future development for patient health information. The low actionability of LLM outputs is likely due to liability constraints that restrict explicit medical recommendations (Jung 2025). It is also influenced by training data that focuses more on providing information than on directive, patient-centered content (AlSammarraie and Househ 2025). Safety and alignment training processes (e.g., reinforcement learning from human feedback) also make LLMs more cautious, so they rarely provide concrete treatment recommendations (Chen et al. 2025). Instead, they favor explanatory and risk-avoiding responses to prevent potentially harmful advice (Chen et al. 2025). Although this caution makes the outputs safer, it also makes them less actionable and less useful for supporting clinical decisions.

LLMs outputs were generally difficult to read (median FRES: 34.5), far below the recommended FRES ≥ 70 (6th-8th grade reading level) for patient materials (Weiss 2003). Our findings align with previous studies showing that LLMs tend to use linguistically complex language (Pan et al. 2023; Yeo et al. 2023; Davis et al. 2024; Hershenhouse et al. 2025). To serve as useful sources of patient information, LLMs answers must be easily understandable (Hanish et al. 2023). This is particularly important since acquiring health information plays a crucial role in strengthening health literacy (Hancı et al. 2024).

The low overall risk of misinformation is encouraging. While previous studies in healthcare contexts have reported that LLMs often provide partially inaccurate or incomplete information (Pan et al. 2023; Yeo et al. 2023; Davis et al. 2024), our findings are in accordance with a recent study that also found LLMs to generally provide reliable cancer-related information (Pan et al. 2023) suggesting that the evaluated LLMs are now producing more reliable responses. This improvement may reflect advances in model architecture, training data, and retrieval strategies. However, this should not distract from the fact that individual inaccurate or incomplete patient information can have serious consequences, particularly for vulnerable patient groups such as HNC patients. A recent study demonstrated that the performance of LLMs depends on how specific the prompt is (Lee and Shin 2024). When prompts are vague or too general, the models often produce answers that are less accurate, less useful, or even incorrect (Lee and Shin 2024). The understandability, actionability, and readability of LLM responses varied across different question categories, highlighting that the choice of LLM can influence the usefulness of patient information on different health-related aspects of HNC (Table 4). Certain categories, such as side effects and nutrition, exhibited comparatively lower PEMAT understandability scores. However, all categories still exceeded the 70% benchmark (Shoemaker et al. 2020; Mani et al. 2021; Shan et al. 2023), indicating overall good comprehensibility. Actionability was very low in the categories of side effects, dental care, gustatory dysfunction, and nutrition (median PEMAT score: 0), indicating that LLMs, while informative, rarely provide concrete guidance where active patient support is needed. Consequently, these responses are unsuitable as a sole resource, and patients require additional medical or dental consultation.

The good performance of the two LLMs Perplexity and Gemini–2.5 Flash can be attributed in part to their training on a broader and more curated data basis. Perplexity relies on retrieval augmentation, combining LLM outputs with web search and automatically citing sources (Xiong et al. 2024), most likely explaining higher DISCERN scores. Gemini–2.5 Flash is a multimodal model trained on a very large and up-to-date dataset (Rane et al. 2024). In contrast, the weaker performance of Microsoft Copilot may be explained by less optimized training data or insufficient emphasis on patient-centered language (Pan et al. 2023; Hancı et al. 2024). Interpretations are limited, as the exact training data, fine-tuning procedures, and weighting of the LLMs are not transparently disclosed.

A main limitation of our study is that only German-language questions were examined, which limits generalizability to other languages and non-German-speaking patients. Since many LLMs are primarily trained and developed using English-language data and within an English-speaking cultural context, their performance may differ in other languages and cultural settings, further limiting the applicability of these results (Cuskley et al. 2024). Additionally, data were collected in June 2025, making the results time-sensitive given the rapid development of LLMs. As no statistics were available for the top 10 questions related to oral health and side effects in HNC, the questions were obtained directly from LLMs (ChatGPT–GPT-4-turbo and Gemini–2.5 Flash), meaning that not all frequently asked patient questions may have been captured. This method introduces a risk of circular reasoning, and basing the questions on real patients, or input from expert clinician panels would likely have enhanced the study’s clinical relevance. Responses were evaluated by two experts, while including perspectives from patients, oncology nurses, or health literacy experts could have further strengthened the assessment of subjective metrics such as understandability and actionability. Moreover, the study design does not allow for definitive conclusions regarding the use of LLMs in HNC patient education. Important questions remain open, including the impact of LLMs on oral health outcomes and their potential influence on patients’ treatment decisions.

Nevertheless, the study has several strengths. It provides a systematic and comparative evaluation of eight LLMs on clinically relevant queries, using methods previously described in the literature (Pan et al. 2023; Hancı et al. 2024). It also assesses multiple parameters, including reliability and quality, understandability and actionability, and readability, using validated instruments such as DISCERN (Charnock et al. 1999), PEMAT (Shoemaker et al. 2020), and FRES (Flesch 1948; Amstad 1978).

Overall, the findings suggest that LLMs vary in quality, readability, and actionability when addressing oral health questions in patients with HNC. Future research should include multilingual evaluations, assess more complex clinical scenarios, and incorporate patient-centered validation to evaluate understandability and usefulness. Furthermore, standardized benchmarks for actionability and readability in oncology should be developed, and integration into verified medical platforms under clinical supervision is recommended to ensure both utility and patient safety.

## Conclusion

Although the overall risk of misinformation was low, observed shortcomings in actionability underscore the necessity for cautious and supervised integration of LLMs into patient education for HNC. Furthermore, LLMs have the potential to support patient education by providing responses to complex medical questions.

## Supplementary Information

Below is the link to the electronic supplementary material.

Supplementary Material 1

## Data Availability

All data generated or analyzed during this study are included in this article. Further enquiries can be directed to the corresponding author.

## References

[CR1] AlSammarraie A, Househ M (2025) The use of large Language models in generating patient education materials: a scoping review. Acta Inf Med 33(1):4–10. 10.5455/aim.2024.33.4-1010.5455/aim.2024.33.4-10PMC1198633740223858

[CR2] Amstad T (1978) Wie verständlich sind unsere Zeitungen? Studenten-Schreib-Service, Zurich

[CR3] Bhandari S, Soni BW, Bahl A, Ghoshal S (2020) Radiotherapy-induced oral morbidities in head and neck cancer patients. Spec Care Dentist 40:238–250. 10.1111/scd.1246932378765 10.1111/scd.12469

[CR4] Biswas SS (2023) Role of chat GPT in public health. Ann Biomed Eng 51(5):868–869. 10.1007/s10439-023-03172-736920578 10.1007/s10439-023-03172-7

[CR5] Cassidy JT, Baker JF (2016) Orthopaedic patient information on the world wide web: an essential review. J Bone Joint Surg Am 98(4):325–338. 10.2106/jbjs.N.0118926888683 10.2106/JBJS.N.01189

[CR6] Charnock D, Shepperd S, Needham G, Gann R (1999) DISCERN: an instrument for judging the quality of written consumer health information on treatment choices. J Epidemiol Commun Health 53(2):105–111. 10.1136/jech.53.2.10510396471 10.1136/jech.53.2.105PMC1756830

[CR7] Chen S, Gao M, Sasse K, Hartvigsen T, Anthony B, Fan L, Aerts H, Gallifant J, Bitterman DS (2025) When helpfulness backfires: LLMs and the risk of false medical information due to sycophantic behavior. NPJ Digit Med 8(1):605. 10.1038/s41746-025-02008-z41107408 10.1038/s41746-025-02008-zPMC12534679

[CR8] Cicchetti DV (1994) Guidelines, criteria, and rules of thumb for evaluating normed and standardized assessment instruments in psychology. Psychol Assess 6(4):284. 10.1037/1040-3590.6.4.284

[CR9] Colevas AD, Cmelak AJ, Pfister DG, Spencer S, Adkins D, Birkeland AC, Brizel DM, Busse PM, Caudell JJ, Durm G, Fakhry C, Galloway T, Geiger JL, Gillison ML, Glastonbury C, Haddad RI, Hicks WL, Hitchcock YJ, Jimeno A, Juloori A, Kase M, Leizman D, Maghami E, Mell LK, Mittal BB, Pinto HA, Price K, Rocco JW, Rodriguez CP, Schwartz D, Shah JP, Sher D, John MS, Wang H, Weinstein G, Worden F, Bruce JY, Yom SS, Zhen W, Montgomery S, Darlow SD (2025) NCCN Guidelines^®^ insights: head and neck Cancers, version 2.2025. J Natl Compr Canc Netw 23(2):2–11. 10.6004/jnccn.2025.000739938471 10.6004/jnccn.2025.0007

[CR10] Cuskley C, Woods R, Flaherty M (2024) The limitations of large language models for understanding human language and cognition. Open Mind (Camb) 8:1058–1083. 10.1162/opmi_a_0016039229609 10.1162/opmi_a_00160PMC11370970

[CR11] Davis RJ, Ayo-Ajibola O, Lin ME, Swanson MS, Chambers TN, Kwon DI, Kokot NC (2024) Evaluation of oropharyngeal cancer information from revolutionary artificial intelligence chatbot. Laryngoscope 134(5):2252–2257. 10.1002/lary.3119137983846 10.1002/lary.31191

[CR12] Derevianko A, Pizzoli SFM, Pesapane F, Rotili A, Monzani D, Grasso R, Cassano E, Pravettoni G (2023) The use of artificial intelligence (AI) in the radiology field: what is the state of Doctor-Patient communication in cancer diagnosis? Cancers (Basel) 15(2). 10.3390/cancers1502047010.3390/cancers15020470PMC985682736672417

[CR13] Doctor R, Padhya T, Mifsud M, Nickel C (2024) A systematic review of approaches to dental care in head and neck cancer patients. Oral Oncol Rep 9:100205. 10.1016/j.oor.2024.100205

[CR14] Eggmann F, Weiger R, Zitzmann NU, Blatz MB (2023) Implications of large Language models such as ChatGPT for dental medicine. J Esthet Restor Dent 35(7):1098–1102. 10.1111/jerd.1304637017291 10.1111/jerd.13046

[CR15] Elad S, Cheng KKF, Lalla RV, Yarom N, Hong C, Logan RM, Bowen J, Gibson R, Saunders DP, Zadik Y, Ariyawardana A, Correa ME, Ranna V, Bossi P (2020) MASCC/ISOO clinical practice guidelines for the management of mucositis secondary to cancer therapy. Cancer 126(19):4423–4431. 10.1002/cncr.3310032786044 10.1002/cncr.33100PMC7540329

[CR16] Epstein JB, Thariat J, Bensadoun RJ, Barasch A, Murphy BA, Kolnick L, Popplewell L, Maghami E (2012) Oral complications of cancer and cancer therapy: from cancer treatment to survivorship. CA Cancer J Clin 62(6):400–422. 10.3322/caac.2115722972543 10.3322/caac.21157

[CR17] Flesch R (1948) A new readability yardstick. J Appl Psychol 32(3):221–233. 10.1037/h005753218867058 10.1037/h0057532

[CR18] Gouvêa Vasconcellos AF, Palmier NR, Ribeiro ACP, Normando AGC, Morais-Faria K, Gomes-Silva W, Vechiato Filho AJ, de Goes MF, Paes Leme AF, Brandão TB, Lopes MA, Marsh PD, Santos-Silva AR (2020) Impact of clustering oral symptoms in the pathogenesis of radiation caries: a systematic review. Caries Res 54(2):113–126. 10.1159/00050487831962337 10.1159/000504878

[CR19] Gül Ş, Erdemir İ, Hanci V, Aydoğmuş E, Erkoç YS (2024) How artificial intelligence can provide information about subdural hematoma: assessment of readability, reliability, and quality of ChatGPT, BARD, and perplexity responses. Med (Baltim) 103(18):e38009. 10.1097/MD.000000000003800910.1097/MD.0000000000038009PMC1106265138701313

[CR20] Hancı V, Ergün B, Gül Ş, Uzun Ö, Erdemir İ, Hancı FB (2024) Assessment of readability, reliability, and quality of ChatGPT^®^, BARD^®^, Gemini^®^, Copilot^®^, Perplexity^®^ responses on palliative care. Med (Baltim) 103(33):e39305. 10.1097/MD.000000000003930510.1097/MD.0000000000039305PMC1133273839151545

[CR21] Hanish SJ, Cherian N, Baumann J, Gieg SD, DeFroda S (2023) Reducing the use of complex words and reducing sentence length to < 15 words improves readability of patient education materials regarding sports medicine knee injuries. Arthrosc Sports Med Rehabil 5(1):e1–e9. 10.1016/j.asmr.2022.10.00436866291 10.1016/j.asmr.2022.10.004PMC9971903

[CR22] Hershenhouse JS, Mokhtar D, Eppler MB, Rodler S, Storino Ramacciotti L, Ganjavi C, Hom B, Davis RJ, Tran J, Russo GI, Cocci A, Abreu A, Gill I, Desai M, Cacciamani GE (2025) Accuracy, readability, and understandability of large Language models for prostate cancer information to the public. Prostate Cancer Prostatic Dis 28(2):394–399. 10.1038/s41391-024-00826-y38744934 10.1038/s41391-024-00826-yPMC12106072

[CR23] Hryciw BN, Fortin Z, Ghossein J, Kyeremanteng K (2023) Doctor-patient interactions in the age of AI: navigating innovation and expertise. Front Med (Lausanne) 10:1241508. 10.3389/fmed.2023.124150837711734 10.3389/fmed.2023.1241508PMC10498385

[CR24] Huo B, Collins GS, Chartash D, Thirunavukarasu AJ, Flanagin A, Iorio A, Cacciamani G, Chen X, Liu N, Mathur P, Chan AW, Laine C, Pacella D, Berkwits M, Antoniou SA, Camaradou JC, Canfield C, Mittelman M, Feeney T, Loder EW, Agha R, Saha A, Mayol J, Sunjaya A, Harvey H, Ng JY, McKechnie T, Lee Y, Verma N, Stiglic G, McCradden M, Ramji K, Boudreau V, Ortenzi M, Meerpohl JJ, Vandvik PO, Agoritsas T, Samuel D, Frankish H, Anderson M, Yao X, Loeb S, Lokker C, Liu X, Guallar E, Guyatt GH (2025) Reporting guideline for chatbot health advice studies: the CHART statement. JAMA Netw Open 8(8):e2530220. 10.1001/jamanetworkopen.2025.3022040747871 10.1001/jamanetworkopen.2025.30220

[CR26] Jabbour J, Milross C, Sundaresan P, Ebrahimi A, Shepherd HL, Dhillon HM, Morgan G, Ashford B, Abdul-Razak M, Wong E, Veness M, Palme CE, Froggatt C, Cohen R, Ekmejian R, Tay J, Roshan D, Clark JR (2017) Education and support needs in patients with head and neck cancer: a multi-institutional survey. Cancer 123(11):1949–1957. 10.1002/cncr.3053528081302 10.1002/cncr.30535

[CR25] Jabbour J, Dhillon HM, Shepherd HL, Sundaresan P, Milross C, Clark JR (2022) A web-based comprehensive head and neck cancer patient education and support needs program: usability testing. Health Inf J 0(0):14604582221087128. 10.1177/1460458222108712810.1177/1460458222108712835362344

[CR27] Jung KH (2025) Large Language models in medicine: clinical applications, technical challenges, and ethical considerations. Healthc Inf Res 31(2):114–124. 10.4258/hir.2025.31.2.11410.4258/hir.2025.31.2.114PMC1208643840384063

[CR28] Lee JH, Shin J (2024) How to optimize prompting for large Language models in clinical research. Korean J Radiol 25(10):869–873. 10.3348/kjr.2024.069539344543 10.3348/kjr.2024.0695PMC11444847

[CR29] Loeb S, Sengupta S, Butaney M, Macaluso JN Jr., Czarniecki SW, Robbins R, Braithwaite RS, Gao L, Byrne N, Walter D, Langford A (2019) Dissemination of misinformative and biased information about prostate cancer on YouTube. Eur Urol 75(4):564–567. 10.1016/j.eururo.2018.10.05630502104 10.1016/j.eururo.2018.10.056

[CR30] Machiels JP, René Leemans C, Golusinski W, Grau C, Licitra L, Gregoire V (2020) Squamous cell carcinoma of the oral cavity, larynx, oropharynx and hypopharynx: EHNS-ESMO-ESTRO clinical practice guidelines for diagnosis, treatment and follow-up. Ann Oncol 31(11):1462–1475. 10.1016/j.annonc.2020.07.01133239190 10.1016/j.annonc.2020.07.011

[CR31] Mani NS, Ottosen T, Fratta M, Yu F (2021) A health literacy analysis of the consumer-oriented COVID-19 information produced by ten state health departments. J Med Libr Assoc 109(3):422–431. 10.5195/jmla.2021.116534629971 10.5195/jmla.2021.1165PMC8485956

[CR32] Meyrowitsch DW, Jensen AK, Sørensen JB, Varga TV (2023) AI chatbots and (mis)information in public health: impact on vulnerable communities. Front Public Health 11:1226776. 10.3389/fpubh.2023.122677638026315 10.3389/fpubh.2023.1226776PMC10644115

[CR33] Nekhlyudov L, Lacchetti C, Davis NB, Garvey TQ, Goldstein DP, Nunnink JC, Ninfea JIR, Salner AL, Salz T, Siu LL (2017) Head and neck cancer survivorship care guideline: American society of clinical oncology clinical practice guideline endorsement of the American cancer society guideline. J Clin Oncol 35(14):1606–1621. 10.1200/JCO.2016.71.847828240970 10.1200/JCO.2016.71.8478

[CR34] Omiye JA, Lester JC, Spichak S, Rotemberg V, Daneshjou R (2023) Large Language models propagate race-based medicine. NPJ Digit Med 6(1):195. 10.1038/s41746-023-00939-z37864012 10.1038/s41746-023-00939-zPMC10589311

[CR35] Pan A, Musheyev D, Bockelman D, Loeb S, Kabarriti AE (2023) Assessment of artificial intelligence chatbot responses to top searched queries about cancer. JAMA Oncol 9(10):1437–1440. 10.1001/jamaoncol.2023.294737615960 10.1001/jamaoncol.2023.2947PMC10450581

[CR36] Park KU, Lipsitz S, Dominici LS, Lynce F, Minami CA, Nakhlis F, Waks AG, Warren LE, Eidman N, Frazier J, Hernandez L, Leslie C, Rafte S, Stroud D, Weissman JS, King TA, Mittendorf EA (2025) Generative artificial intelligence as a source of breast cancer information for patients: proceed with caution. Cancer 131(1):e35521. 10.1002/cncr.3552139211977 10.1002/cncr.35521

[CR37] Peterson DE, Koyfman SA, Yarom N, Lynggaard CD, Ismaila N, Forner LE, Fuller CD, Mowery YM, Murphy BA, Watson E, Yang DH, Alajbeg I, Bossi P, Fritz M, Futran ND, Gelblum DY, King E, Ruggiero S, Smith DK, Villa A, Wu JS, Saunders D (2024) Prevention and management of osteoradionecrosis in patients with head and neck cancer treated with radiation therapy: ISOO-MASCC-ASCO guideline. J Clin Oncol 42(16):1975–1996. 10.1200/jco.23.0275038691821 10.1200/JCO.23.02750

[CR38] Pfister DG, Spencer S, Adelstein D, Adkins D, Anzai Y, Brizel DM, Bruce JY, Busse PM, Caudell JJ, Cmelak AJ, Colevas AD, Eisele DW, Fenton M, Foote RL, Galloway T, Gillison ML, Haddad RI, Hicks WL, Hitchcock YJ, Jimeno A, Leizman D, Maghami E, Mell LK, Mittal BB, Pinto HA, Ridge JA, Rocco JW, Rodriguez CP, Shah JP, Weber RS, Weinstein G, Witek M, Worden F, Yom SS, Zhen W, Burns JL, Darlow SD (2020) Head and neck Cancers, version 2.2020, NCCN clinical practice guidelines in oncology. J Natl Compr Canc Netw 18(7):873–898. 10.6004/jnccn.2020.003132634781 10.6004/jnccn.2020.0031

[CR39] Pradhan SK, Furian M, Todeschini G, Schürer Q, Wang X, Chen B, Li Y, Gantenbein AR (2025) Daith Piercing, a social media hype on Youtube for the treatment of migraine? A systematic video analysis. Health Sci Rep 8(6):e70880. 10.1002/hsr2.7088040503346 10.1002/hsr2.70880PMC12153007

[CR40] Qiu J, Li L, Sun J, Peng J, Shi P, Zhang R, Dong Y, Lam K, Lo FP, Xiao B, Yuan W, Wang N, Xu D, Lo B (2023) Large AI models in health informatics: applications, Challenges, and the future. IEEE J Biomed Health Inf 27(12):6074–6087. 10.1109/jbhi.2023.331675010.1109/JBHI.2023.331675037738186

[CR41] Rane N, Choudhary S, Rane J (2024) Gemini versus chatgpt: applications, performance, architecture, capabilities, and implementation. J App Artif Intell 5(1):69–93. 10.48185/jaai.v5i1.1052

[CR42] Rose AM, Helgeson ES, Valentino KC, Lalla RV, Treister NS, Schmidt BL, Patton LL, Lin A, Brennan MT, Sollecito TP (2025) The impact of medications on salivary flow and oral health-related quality of life in postradiation head and neck cancer patients: results of the OraRad study. Oral Surg Oral Med Oral Pathol Oral Radiol 140(5):577–586. 10.1016/j.oooo.2025.06.01940784870 10.1016/j.oooo.2025.06.019PMC13050463

[CR43] Scheirer CJ, Ray WS, Hare N (1976) The analysis of ranked data derived from completely randomized factorial designs. Biometrics 32(2):429–434. 10.2307/2529511 953139

[CR44] Schöll P (2025) Berechnung—Fleschindex [Calculation—Flesch reading-ease score]. https://fleschindex.de/berechnen/. Accessed 15 Sept 2025

[CR45] Shan Y, Ji M, Dong Z, Xing Z, Wang D, Cao X (2023) The Chinese version of the patient education materials assessment tool for printable materials: Translation, Adaptation, and validation study. J Med Internet Res 25:e39808. 10.2196/3980837200085 10.2196/39808PMC10236277

[CR46] Shoemaker SJ, Wolf MS, Brach C (2020) The patient education materials assessment tool (PEMAT) and user’s guide. https://www.ahrq.gov/health-literacy/patient-education/pemat.html. Accessed 15 Sept 2025

[CR47] Shrout PE, Fleiss JL (1979) Intraclass correlations: uses in assessing rater reliability. Psychol Bull 86(2):420–428. 10.1037/0033-2909.86.2.42018839484 10.1037//0033-2909.86.2.420

[CR48] Sivaramakrishnan G, Almuqahwi M, Ansari S, Lubbad M, Alagamawy E, Sridharan K (2025) Assessing the power of AI: a comparative evaluation of large Language models in generating patient education materials in dentistry. BDJ Open 11(1):59. 10.1038/s41405-025-00349-140533491 10.1038/s41405-025-00349-1PMC12177049

[CR49] Sun J, Zhang S, Hou M, Sun Q, Cao F, Zhang Z, Tang G, Wang X, Geng L, Cui L, Chen ZJ (2023) Who can help me? Understanding the antecedent and consequence of medical information seeking behavior in the era of bigdata. Front Public Health 11:1192405. 10.3389/fpubh.2023.119240537790712 10.3389/fpubh.2023.1192405PMC10544578

[CR50] Tong A, Flemming K, McInnes E, Oliver S, Craig J (2012) Enhancing transparency in reporting the synthesis of qualitative research: ENTREQ. BMC Med Res Methodol 12:181. 10.1186/1471-2288-12-18123185978 10.1186/1471-2288-12-181PMC3552766

[CR51] Türe N, Tahir E, Enver N (2025) Readability, understandability, and quality of online education materials and large Language models for retrograde cricopharyngeal muscle dysfunction. Eur Arch Otorhinolaryngol 282(9):4711–4720. 10.1007/s00405-025-09628-x40802099 10.1007/s00405-025-09628-x

[CR52] Uzun O (2023) Assessment of reliability and quality of videos on medial epicondylitis shared on YouTube. Cureus 15(4):e37250. 10.7759/cureus.3725037168186 10.7759/cureus.37250PMC10166569

[CR53] Vedula SS, Ghazi A, Collins JW, Pugh C, Stefanidis D, Meireles O, Hung AJ, Schwaitzberg S, Levy JS, Sachdeva AK (2022) Artificial intelligence methods and artificial intelligence-Enabled metrics for surgical education: a multidisciplinary consensus. J Am Coll Surg 234(6):1181–1192. 10.1097/xcs.000000000000019035703817 10.1097/XCS.0000000000000190PMC10634198

[CR54] Wadhawan R, Datta A, Gogula S, Krishnan A, Yadav DK, Choudhary T (2025) Challenges in chemotherapy for head and neck cancer: a review. Bioinformation 21(2):121–126. 10.6026/97320630021012140322690 10.6026/973206300210121PMC12044184

[CR55] Watson E, Dorna Mojdami Z, Oladega A, Hope A, Glogauer M (2021) Clinical practice guidelines for dental management prior to radiation for head and neck cancer. Oral Oncol 123:105604. 10.1016/j.oraloncology.2021.10560434775180 10.1016/j.oraloncology.2021.105604

[CR56] Weil AG, Bojanowski MW, Jamart J, Gustin T, Lévêque M (2014) Evaluation of the quality of information on the internet available to patients undergoing cervical spine surgery. World Neurosurg 82(1–2):e31-39. 10.1016/j.wneu.2012.11.00310.1016/j.wneu.2012.11.00323142585

[CR57] Weiss BD (2003) Health literacy. Am Med Assoc 253:358

[CR58] Xiong G, Jin Q, Lu Z, Zhang A (2024) Benchmarking retrieval-augmented generation for medicine. Findings of the association for computational linguistics. ACL:6233–6251. 10.18653/v1/2024.findings-acl.372

[CR59] Yeo YH, Samaan JS, Ng WH, Ting PS, Trivedi H, Vipani A, Ayoub W, Yang JD, Liran O, Spiegel B, Kuo A (2023) Assessing the performance of ChatGPT in answering questions regarding cirrhosis and hepatocellular carcinoma. Clin Mol Hepatol 29(3):721–732. 10.3350/cmh.2023.008936946005 10.3350/cmh.2023.0089PMC10366809

