# Supplementary material for: Performance of large language models in reporting oral health concerns and side effects in head and neck cancer: a comparative study
Source: J Cancer Res Clin Oncol. 2025 Dec 20;152(1):17. doi: 10.1007/s00432-025-06400-w (PMC12718290; doi:10.1007/s00432-025-06400-w)
Supplement: Supplementary file 1 — Supplementary Material 1 [file 432_2025_6400_MOESM1_ESM.docx]

**Supplemental material**

**Performance of large language models in reporting oral health concerns and side effects in head and neck cancer: a comparative study**

Journal of Cancer Research and Clinical Oncology

Jonas Rast^a^, Susanne Wiegand^a^, Jana Biermann^b^, Annette Wiegand^b^, Felix Marschner^b^

^a^University Hospital Schleswig-Holstein, Department of Otorhinolaryngology, Head and Neck Surgery, Arnold-Heller-Straße 3, 24105 Kiel, Germany

^b^University Medical Center Göttingen, Department of Preventive Dentistry, Periodontology and Cariology, Robert-Koch-Str. 40, 37075 Göttingen, Germany

**Corresponding author**

Dr. Felix Marschner
Department of Preventive Dentistry, Periodontology and Cariology

University Medical Center Göttingen
Robert-Koch-Str. 40, 37075 Göttingen, Germany
E-Mail: felix.marschner@med.uni-goettingen.de

Phone: +49 551 39-60870

Fax: +49 551 39-60869

Supplemental Table S1. Categorization of patient-centered questions on oral health and oral side effects of head and neck cancer therapy generated by ChatGPT–GPT-4-turbo (OpenAI) and Gemini–2.5 Flash (Google).

| **Category** | **Original question translated in English (question number)** | **Large language model** | **Examiner** | **Original question in German** |
| --- | --- | --- | --- | --- |
| Dental and oral hygiene | How important is oral hygiene before, during, and after the treatment? (Q1) | Gemini–2.5 Flash | F.M. | Wie wichtig ist die Mundhygiene vor, während und nach der Behandlung? |
|  | What can I do to keep my mouth clean and healthy during and after the treatment? (Q2) | Gemini–2.5 Flash | J.R. | Was kann ich tun, um meinen Mund während und nach der Behandlung sauber und gesund zu halten |
|  | What dental care products should I use during the therapy? (Q3) | ChatGPT–GPT-4-turbo | J.R. | Welche Zahnpflegeprodukte sollte ich während der Therapie verwenden? |
|  | What role does professional dental cleaning play before, during, and after the therapy? (Q4) | ChatGPT–GPT-4-turbo | F.M. | Welche Rolle spielt die professionelle Zahnreinigung vor, während und nach der Therapie? |
|  | Can I brush my teeth during the therapy, and which toothpaste and mouthwash should I use? (Q5) | Gemini–2.5 Flash | F.M. | Kann ich meine Zähne während der Therapie putzen, und welche Zahnpasta und Mundspülung sollte ich verwenden? |
|  | How do I best take care of my mouth during the therapy? (Q6) | ChatGPT–GPT-4-turbo | F.M. | Wie pflege ich meinen Mund während der Therapie am besten? |
|  | How can I protect my oral mucosa during the therapy? (Q7) | ChatGPT–GPT-4-turbo | J.R. | Wie kann ich meine Mundschleimhaut während der Therapie schützen? |
|  | How can I maintain my oral health in the long term after tumor therapy? (Q8) | ChatGPT–GPT-4-turbo | F.M. | Wie kann ich langfristig meine Mundgesundheit nach der Tumortherapie erhalten? |
| Side effect | What are the most common oral side effects of a head and neck tumor therapy (chemotherapy, radiation therapy, surgery)? (Q9) | Gemini–2.5 Flash | F.M. | Was sind die häufigsten oralen Nebenwirkungen einer Kopf-Hals-Tumor-Therapie (Chemotherapie, Strahlentherapie, Operation)? |
|  | What side effects does radiation or chemotherapy have on oral health? (Q10) | ChatGPT–GPT-4-turbo | J.R. | Welche Nebenwirkungen hat eine Strahlen- oder Chemotherapie auf die Mundgesundheit? |
|  | What side effects can radiation or chemotherapy cause in the oral area? (Q11) | ChatGPT–GPT-4-turbo | F.M. | Welche Nebenwirkungen kann eine Strahlen- oder Chemotherapie im Mundbereich verursachen? |
|  | What oral side effects can the therapy of head and neck tumors cause? (Q12) | Gemini–2.5 Flash | J.R. | Welche oralen Nebenwirkungen kann die Therapie von Kopf-Hals-Tumoren verursachen? |
|  | What long-term oral side effects can occur, and how are they treated? (Q13) | Gemini–2.5 Flash | J.R. | Welche langfristigen oralen Nebenwirkungen können auftreten, und wie werden sie behandelt? |

Supplemental Table S1. Continued.

| **Category** | **Original question translated in English (question number)** | **Large language model** | **Examiner** | **Original question in German** |
| --- | --- | --- | --- | --- |
|  | How long do the side effects in the mouth last after the therapy? (Q14) | ChatGPT–GPT-4-turbo | J.R. | Wie lange halten die Nebenwirkungen im Mund nach der Therapie an? |
| Dental care | When should I see my dentist, and is special dental care necessary? (Q15) | Gemini–2.5 Flash | F.M. | Wann sollte ich meinen Zahnarzt aufsuchen, und ist eine spezielle zahnärztliche Betreuung notwendig? |
|  | Should I see a dentist before, during, and after the therapy? (Q16) | Gemini–2.5 Flash | J.R. | Sollte ich vor, während und nach der Therapie einen Zahnarzt aufsuchen? |
|  | When should I go to the dentist or to oral and maxillofacial surgery before starting tumor therapy? (Q17) | ChatGPT–GPT-4-turbo | F.M. | Wann sollte ich vor Beginn der Tumortherapie zum Zahnarzt oder zur Mund-Kiefer-Gesichtschirurgie? |
|  | Do I have to go to the dentist before starting the therapy? (Q18) | ChatGPT–GPT-4-turbo | J.R. | Muss ich vor Beginn der Therapie zum Zahnarzt? |
| Xerostomia | How can I relieve dry mouth (xerostomia)? (Q19) | Gemini–2.5 Flash | F.M. | Wie kann ich Mundtrockenheit (Xerostomie) lindern? |
|  | What can I do against severe dry mouth (xerostomia)? (Q20) | ChatGPT–GPT-4-turbo | F.M. | Was kann ich gegen die starke Mundtrockenheit (Xerostomie) tun? |
|  | What can I do against severe dry mouth (xerostomia)? (Q21) | ChatGPT–GPT-4-turbo | J.R. | Was kann ich gegen starke Mundtrockenheit (Xerostomie) tun? |
|  | Will my mouth be dry, and what can I do about it? (Q22) | Gemini–2.5 Flash | J.R. | Wird mein Mund trocken sein, und was kann ich dagegen tun? |
| Dental complications | What happens to my teeth after radiation therapy? Is there a risk of cavities or tooth loss? (Q23) | ChatGPT–GPT-4-turbo | F.M. | Was passiert mit meinen Zähnen nach der Strahlentherapie? Besteht die Gefahr von Karies oder Zahnverlust? |
|  | Can the therapy permanently damage my teeth or lead to tooth loss? (Q24) | Gemini–2.5 Flash | F.M. | Kann die Therapie meine Zähne dauerhaft schädigen oder zu Zahnverlust führen? |
|  | Can the treatment damage my teeth or lead to tooth loss? (Q25) | Gemini–2.5 Flash | J.R. | Kann die Behandlung meine Zähne schädigen oder zu Zahnverlust führen? |
| Gustatory dysfunction | Will my sense of taste be affected, and is it reversible? (Q26) | Gemini–2.5 Flash | J.R. | Wird mein Geschmackssinn beeinträchtigt, und ist das reversibel? |
|  | Why does my taste change during therapy? (Q27) | ChatGPT–GPT-4-turbo | J.R. | Warum verändert sich mein Geschmack während der Therapie? |

Supplemental Table S1. Continued.

| **Category** | **Original question translated in English (question number)** | **Large language model** | **Reviewer** | **Original question in German** |
| --- | --- | --- | --- | --- |
|  | What can I do if my sense of taste is affected or everything tastes "different"? (Q28) | Gemini–2.5 Flash | F.M. | Was kann ich tun, wenn mein Geschmackssinn beeinträchtigt ist oder alles "anders" schmeckt? |
| Mucositis | What helps with painful inflammation of the oral mucosa (mucositis)?  (Q29) | Gemini–2.5 Flash | F.M. | Was hilft bei schmerzhaften Mundschleimhautentzündungen (Mukositis)? |
|  | How do I deal with inflammation of the oral mucosa (mucositis) that causes pain? (Q30) | Gemini–2.5 Flash | J.R. | Wie gehe ich mit Mundschleimhautentzündungen (Mukositis) um, die Schmerzen verursachen? |
|  | What is mucositis and how can I treat or prevent it? (Q31) | ChatGPT–GPT-4-turbo | F.M. | Was ist eine Mukositis und wie kann ich sie behandeln oder vermeiden? |
| Nutrition | What dietary recommendations are there for oral side effects?  (Q32) | Gemini–2.5 Flash | F.M. | Welche Ernährungsempfehlungen gibt es bei oralen Nebenwirkungen? |
|  | Do I have to change my diet, and do I possibly need a feeding tube? (Q33) | Gemini–2.5 Flash | J.R. | Muss ich meine Ernährung umstellen, und brauche ich möglicherweise eine Ernährungssonde? |
|  | What am I allowed to eat or drink during therapy with a sensitive mouth? (Q34) | ChatGPT–GPT-4-turbo | F.M. | Was darf ich essen oder trinken während der Therapie bei empfindlichem Mund? |
| Oral pain | How can I relieve pain in the mouth and throat during therapy? (Q35) | ChatGPT–GPT-4-turbo | J.R | Wie kann ich Schmerzen im Mund und Rachen während der Therapie lindern? |
|  | How can I deal with the pain caused by oral side effects? (Q36) | Gemini–2.5 Flash | F.M. | Wie kann ich mit dem Schmerz umgehen, der durch orale Nebenwirkungen verursacht wird? |
|  | How can I relieve pain in the mouth during therapy? (Q37) | ChatGPT–GPT-4-turbo | F.M. | Wie kann ich Schmerzen im Mund während der Therapie lindern? |
| Dysphagia | Do I have difficulties swallowing, and are there therapies for it? (Q38) | Gemini–2.5 Flash | J.R | Habe ich Schwierigkeiten beim Schlucken, und gibt es Therapien dafür? |
| Osteoradio-necrosis | What is osteoradionecrosis of the jaw and how can I prevent it? (Q39) | ChatGPT–GPT-4-turbo | J.R | Was ist eine Osteoradionekrose des Kiefers und wie kann ich sie verhindern? |
| Oral candidiasis | What can I do about fungal infections in the mouth (e.g., thrush)? (Q40) | ChatGPT–GPT-4-turbo | J.R | Was kann ich bei Pilzinfektionen im Mund tun (z. B. Soor)? |
